# *Fusarium Graminearum* Growth Inhibition Due to Glucose Starvation Caused by Osthol

**DOI:** 10.3390/ijms9030371

**Published:** 2008-03-14

**Authors:** Zhiqi Shi, Shouguo Shen, Wei Zhou, Fei Wang, Yongjian Fan

**Affiliations:** Food Safety Research and Service institute, Jiangsu Academy of Agricultural Sciences, Nanjing 210014, P.R. China; E-mails: chenhao@jaas.ac.cn; zhouwei@jaas.ac.cn; lyq@jaas.ac.cn; wangcm@jaas.ac.cn

**Keywords:** *Fusarium graminearum*, osthol, inhibition, glucose starvation, chitinase activity

## Abstract

The effects of osthol, a plant coumarin, on morphology, sugar uptake and cell wall components of *Fusarium graminearum* were examined *in vitro* by electron microscopy,^14^C-labelling and enzyme activity detection. The results revealed that osthol could inhibit the hypha growth of *F. graminearum* by decreasing hyphal absorption to reducing sugar. After treatment with 100 μg·mL^−1^ osthol for 24 h, many hyphal fragments of *F. graminearum* appeared. Microscopy observation showed that the cell walls of hyphal fragments blurred and the organelles of the cells degraded with the increasing vacuoles. The *N*-acetyl-D-glucosamine contents and chitinase activity both increased when hypha were treated with 100 μg·mL^−1^ osthol, whereas the activity of β-1,6-glucanase remained unchanged. When *F. graminearum* fed with ^14^C glucose was treated with 100 μg·mL^−1^osthol, glucose contents decreased to the lowest level, while the contents in non-osthol treated controls remained unchanged. These results suggested that chitinase activity might be related to glucose starvation under osthol treatment, and that the appearance of hyphae fragments maybe the results of the promoted chitinase activity which itself triggered chitin degradation.

## 1. Introduction

Fusarium head blight (FHB), caused by *Fusarium graminearum* Schwabe (teleomorph: Gibberella zeae), is a major disease of wheat and has resulted in heavy yield losses in many areas of China, the largest wheat producer in the World [1, 2]. In eastern China, the continuous use of benzimidazole fungicides has caused the appearance of fungicide resistant strains in *F. graminearum* and these resistant populations are becoming dominant [3]. Lacking effective resistant cultures, plant disease control in China nowadays still relies mainly on the application of chemical agents. With the application of the PRC Agricultural Product Quality Security law, research and development of novel fungicides with high safety and efficiency has become a hot topic of crop protection research.

Osthol (7-methoxy-8-[3-methylpent-2-enyl] coumarin), a plant coumarin, is isolated from the dried fruits of *Cnidium Monnieri* which have been used as a herbal medicine since ancient times in China [4]. Recent pharmacological studies have revealed its antiallergic [5, 6], antiosteoporotic [7], and anti-inflammatory [8] activities. Its antifungal activity was proven on *Alternaria alternate, A. ergillus* sp., *Cryptococuus neoformans*, etc. [9]. Our previous studies also showed that osthol has a wide antifungal activity, with the EC_50_ values ranging from 21.15 μg·mL^−1^ to 61.62 μg·mL^−1^ against *Botrytis cinerea, Colletotrichum mllsae, Fusarium graminearum, Phytophora capsici, Sclerotinia sclerotiorum* and other phytopathogenic fungi [10]. So, as a natural plant toxin, osthol has been developed into biopesticides in our lab, however, its antifungal mechanism and mode of action against phytopathogens still remains unknown. Here, we investigated the effects of osthol on *F. graminearum*, pathogen causing FHB, and a preliminary mechanism of action was studied and discussed.

## 2. Results and Discussion

### 2.1 Results

#### 2.1.1 Effects of osthol on hyphal growth

**Figure 1.**
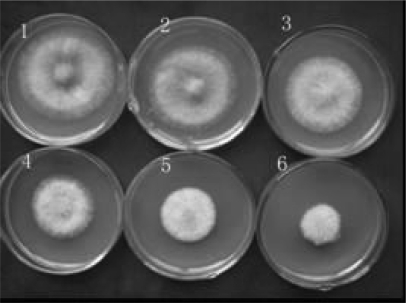
Dose-dependent inhibition of osthol to hyphal diametral growth of *F. graminearum*. Concentration of osthol in PDA medium in Petri plates 1 to 6 was 0, 6.25, 12.5, 25, 50 and 100 μg·mL^−1^, respectively. Experiments were repeated 3 times and each time was done in triplicate.

**Figure 2.**
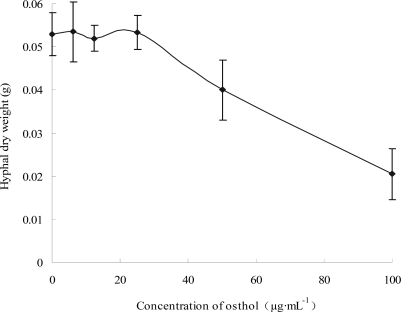
Effect of osthol on hyphal dry weight of *F. graminearum*. Mycelia of *F. graminearum* were harvested from Czapek liquid mediums after 3 days incubation and weighted after drying. Concentration of osthol in Czapek mediums was 0, 6.25, 12.5, 25, 50 and 100 μg·mL^−1^ respectively. Each value is the mean ± S.E. for n=3.

*In vitro* antifungal assays in our previous study had demonstrated that osthol could inhibit the growth of hyphae of *F. graminearum* with its EC_50_ value 56.94 μg·mL^−1^ [10]. Here, the results of disc diffusion test (Figure 1) and biomass assay (Figure 2) further confirmed its inhibitory effects on hyphal growth of *F. graminearum*. With the increase of osthol concentration in PDA medium plates, the inhibitory effect against *F. graminearum* was enhanced through reducing the growth diameter on plates (Figure 1). As shown in Figure 2, osthol began to affect the hyphal growth when its concentration beyond 25 μg·mL^−1^. And the dry weight of hyphae almost behalved when osthol was used at 100 μg·mL^−1^. We did not find hyphal fragment on osthol-treated PDA medium plates. But through microscope observation, we found that more branches appeared from the top edge of hypha treated with osthol (date not shown).

#### 2.2.2 Effects of osthol on hyphae morphology

**Figure 3.**
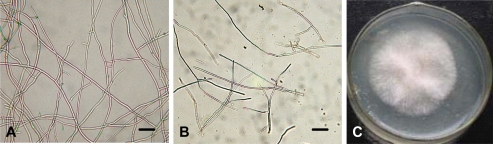
Hyphal fragments appeared in aqueous Czapek culture when *F. graminearum* hyphae were treated with osthol for 24 hours. A, solvent control; B, treatment with 100 μg·mL^−1^ osthol. Bar = 50 μm.

**Figure 4.**
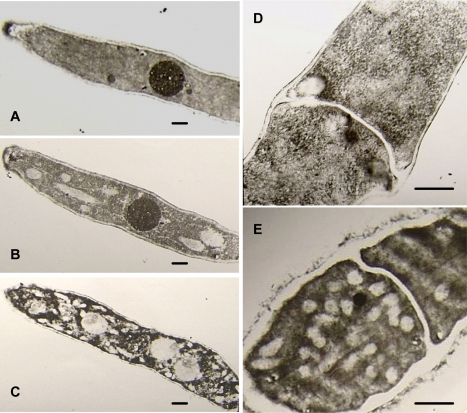
Transmission electron microscope (TEM) analysis showing morphology changes of hyphae of *F. graminearum* after treated with 100 μg·mL^−1^osthol for 24 h. A) blank control; B) and D) solvent control; C) and E) treated with 100 μg·mL^−1^ osthol. Bar = 1 μm.

After treatment with 100 μg·mL^−1^ osthol for 24 hours, the hyphae of *F. graminearum* broke into large fragments (Figure 3B). But these fragments remained actively which could be proved by the result of fragments reinoculation test shown in Figure 3C. Furthermore, the TEM observation showed that the osthol-treated hyphae presented degenerative changes compared to the controls (Figure 4). There are large vesicles appearing in osthol-treated cells (Figure 4C, E) and most of these cell walls were becoming blurring after 24h treatment with 100 μg·mL^−1^ osthol (Figure 4E). In addition, nuclei in cells of blank and solvent controls can be clearly distinguished (Figure 4A, B), however, this was changed in osthol-treated cells. We could not found visible boundary of nucleus (Figure 4C).

#### 2.2.3 Effects of osthol on hyphal glucose absorption

**Figure 5.**
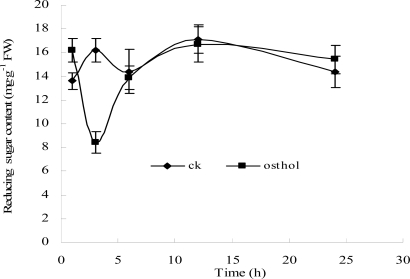
Effect of osthol on hyphal reducing sugar content. “♦” symbolize the reducing sugar content in non-osthol-treated hyphae of *F. graminearum*; “▪” represent the reducing sugar content in hyphae of *F. graminearum* with the treatment of 100 μg·mL^−1^ osthol.

**Figure 6.**
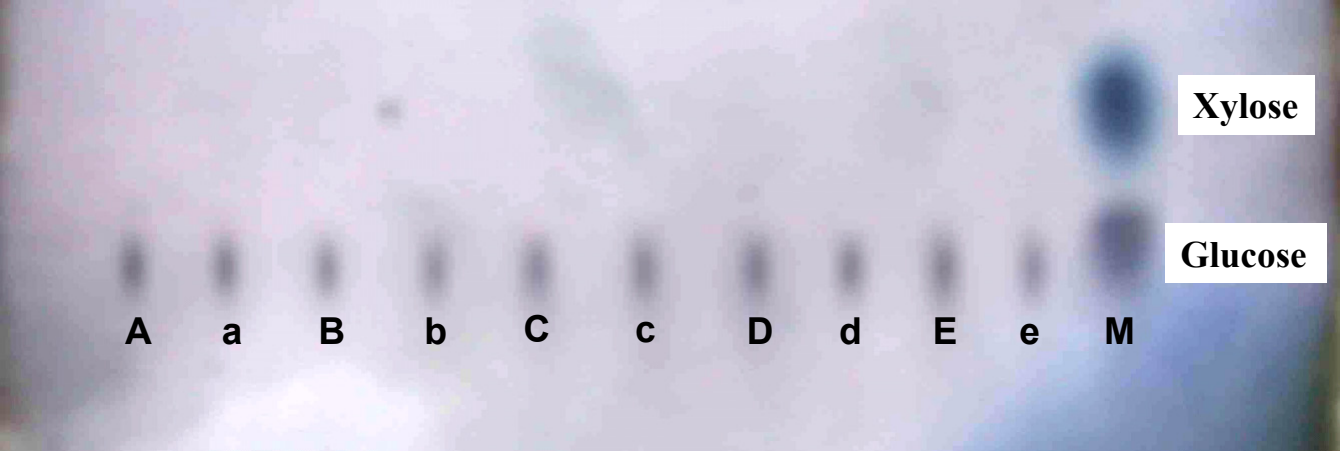
TLC analysis of reducing sugar in hypha of *F. graminearum*. A, B, C, D, E are non-osthol-treated control hyphal reducing sugar spots at 1, 3, 6, 12, 24 h, respectively; a, b, c, d, e are osthol-treated hyphal reducing sugar spots at 1, 3, 6, 12, 24 h, respectively after treated with 100 μg·mL^−1^ osthol; M is the mixture of standard reducing sugars.

**Figure 7.**
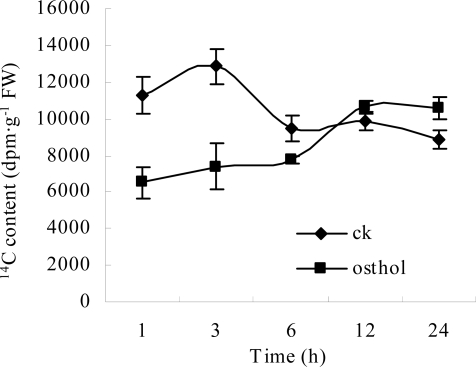
Effect of osthol on ^14^C-labeled glucose absorption by the hyphae of *F. graminearum*. The absorbed ^14^C-labeled glucose in untreated hyphae of *F. graminearum*, ♦;the content of ^14^C-labeled glucose in hyphae of *F. graminearum* with the treatment of 100 μg·mL^−1^ osthol, ▪.

An effect of osthol on hyphal glucose utilization was detected. As shown in Figure 5, within the osthol-treated hyphae reducing sugar levels decreased to the lowest value after 3 h and recovered to the control level 6 h after the treatment. By thin-layer chromatography (TLC) analysis, we found that the reducing sugar in hyphae of *F*. graminearum was mainly composed of glucose (Figure 6). Therefore, ^14^C-labelled glucose was used to investigate the glucose absorption of hyphae. Figure 7 suggests that there is an inhibitory effect of osthol on glucose absorption by hyphae at the beginning stages of the treatment. After longer exposure periods the content of ^14^C-labelled glucose in osthol-treated hyphae increased very slowly, while in the control samples the soluble ^14^C remained at the high beginning level of the experiment indicating the effective absorption of glucose by the hyphae. The decrease of the hyphal soluble ^14^C in control appeared earlier than in the osthol treated samples. These results not only showed a comparatively slow ^14^C-labelled glucose accumulation in osthol-treated hyphae but also indicated that the transformation rate of ^14^C switching from soluble form to insoluble form was slower than the control.

#### 2.2.4 Influence of osthol on cell wall synthesis-related components

**Figure 8.**
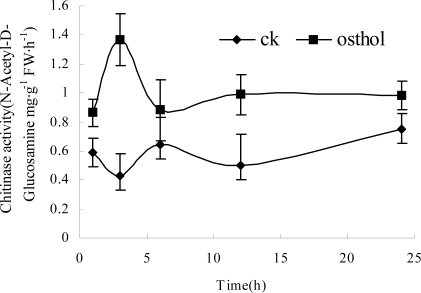
Influence of osthol on chitinase activity in hyphae of *F. graminearum*. Chitinase activity in untreated hyphae of *F. graminearum*, ♦; Chitinase activity in the hyphae of *F. graminearum* with the treatment of 100 μg·mL^−1^ osthol, ▪.

**Figure 9.**
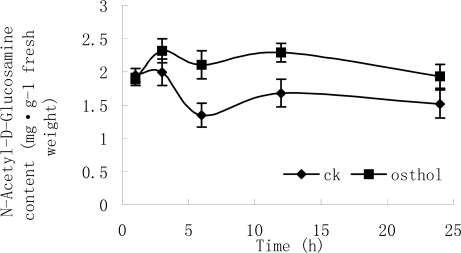
The change of *N*-acetyl-D-glucosamine content in osthol-treated *F. graminearum. N*-Acetyl-D-glucosamine content in untreated hyphae of *F. graminearum*, ♦; *N*-Acetyl-D-glucosamine content in the hyphae of *F. graminearum* with the treatment of 100 μg mL-1 osthol, ▪.

**Figure 10.**
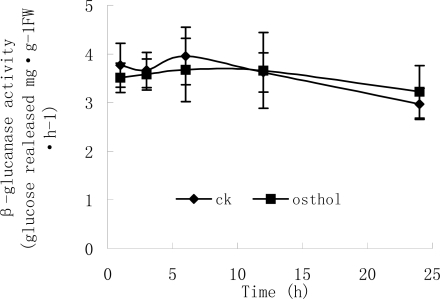
Time course of β-1,6-glucanase activity in osthol-treated hyphae of *F. graminearum*. β-1,6-Glucanase activity in untreated hyphae of *F. graminearum*, ♦; β-1,6-glucanase activity in the hyphae of *F. graminearum* with the treatment of 100 μg·mL^−1^ osthol, ▪.

Morphology observations showed that osthol could cause hyphal cell wall loosening and hyphae breakage. Thereby, components and synthesis-related enzymes of cell wall were examined after hypae were treated with osthol. Figures 8 and 9 show that after treatment with 100 μg·mL^−1^ osthol, chitinase activity and *N*-acetyl-D-glucosamine in osthol-treated hyphae were both higher than in that of the controls. Especially, chitinase activity in osthol-treated hyphae reached the highest level at 3h after the beginning of the treatment. However, β-1,6-glucanase activity showed no obvious difference compared with the control (Figure 10).

### 2.2 Discussion

Osthol displayed a broad-spectrum antifungal activity against phytopathogens [10]. Bioassay demonstrated that osthol showed a high activity against *F. graminearum* (Figures 1–3) and the *in vitro* assays in this study also indicated that osthol could inhibit hyphal growth of *F. graminearum*. Treated with 100 μg·mL^−1^osthol for 24 h, the hyphae of *F. graminearum* growing in aqueous culture broke into fragments and the fragments could develop to colonies after inoculation (Figure 3C). This indicated that the treatment inhibited the growth of the fungi, but was not lethal to them.

The morphological observations showed cytoplasmic vacuolation and blurring of organelles and cell walls after the treatment with osthol (Figure 4). The fungal cell wall consists basically of a complex network of glucan and chitin, playing important roles in several biological processes such as cell shape, morphogenesis, reproduction, cell-cell and cell-matrix interaction, and osmotic, physical protection.

The fungal cell wall is critical for cell viability and pathogenicity. Chitin and β-1,6-glucanase in the cell wall of *F. graminearum* both confer a high mechanical resistance to the cell wall. In previous study, Chaffin and Lopez-Ribot [11] found that chitin was important although only a low content (0.6%–9%) could be found in the wall of *Candida albicans* (mainly in the septum of the hyphae). When the fragments appeared after treated with osthol, the components of cell wall of hyphae and hydrolysis-relative enzymes were measured. The results showed that the chitinase activity increased after the treatment with osthol compared with the control and that *N*-Glc*N*Ac, the chitin unit, remained to a high content in the hyphae. However, β-1,6-glucanase activity did not obviously change (Figure 7–9), which indicated that osthol treatment could not activate the up-regulated expression of glucanase genes in hyphae of *F. graminearum*. So glucan, the important component within cell wall, was not hydrolyzed due to the stabilization of glucanolytic activities. In agreement with this, the results of the TEM experiments (Figure 4) showed clearly that the blurring of cell wall after treated with osthol was mainly caused by the hydrolyzation of chitin in the wall. Hence, the broken hyphae appeared due to the loosing of cell wall together with the water flow pressure in liquid medium (Figure 3B).

As a necessary component of cell wall, chitin could be hydrolyzed when sugar starvation occurred [12]. A previous report had demonstrated that the coumarin compounds could inhibit the glucose absorption by N. *frontalis* [13]. In the present study, decreasing glucose content led to changes to V-shaped form in osthol-treated hyphae of *F. graminearum* suggesting a starvation and a recovery of nutrient (Figure 5). From assay of morphology observation, the large vesicles in osthol-treated hyphae indicated that the treatment of osthol might originate glucose starvation [14]. The origin of the starvation might be the inability of nutrient absorption caused by osthol. The results of the trial about ^14^C-labeled glucose absorption agree with this conclusion (Figure 7). When cells of fungi encounter stress or unfavorable nutrient conditions, the changes of cell were physiologically and morphologically different from the cells growing exponentially. Especially sugar starvation initiates changes in substantial physiological and biochemical processes with the goal of sustaining respiration and other essential metabolic processes [15].

The results also showed that plenty of vacuoles appeared in the osthol-treated hyphae (Figure 4). Autophagy was the major route which cells require enhanced structural compounds degradation and remodeling of components for the maintenance of viability of fungi under starvation conditions [16, 17].

Although basic activity of osthol against the phytopathogen and some changes of physiological activity were reported herein, further detailed research should be focused on direct targets in the phytopathogenic cells.

## 3. Experimental Section

### 3.1 Chemicals and fungal strain

Osthol (97.5%, w/v) was extracted by Plant Protection Institute of Jiangsu Academy of Agricultural Sciences (PPIJA); DMAB (*p*-dimethylaminobenzaldehyde) was purchased from ShangHai SSS Reagent CO., LTD; chitin was purchased from Shanghai Chemical Reagent Corporation; ^14^C-labeled glucose was purchased from China Isotope Corporation; DNS (3,5-dinitrosalicylic acid), ATP (adenosine 5′-triphosphate), β-1,6-glucan, Glc*N*Ac (*N*-acetyl-D-glucosamine), DMSO (dimethyl sulfoxide), Triton X-100, PPO (2,5-diphenyloxazole), POPOP (1,4-bis(5-phenyloxazol-2-yl)benzene) and all other chemicals were obtained from Sigma. Osthol was dissolved in DMSO and prepared as osthol stock solution (20,000 μg·mL^−1^) which was used in the trials. *F. graminearum* was presented by PPIJA and maintained on potato dextrose agar (PDA) medium at 4 °C.

### 3.2 Antifungal activity assays

Antifungal activity of osthol against *F. graminearum* was determined by inhibition of radial growth and biomass of the fungus *in vitro*, respectively. The disc diffusion assay was performed in radiation sterilized (^14^C-labeled glucose) petri plates of 90 mm in diameter. Osthol stock solution was added to autoclaved PDA mediums to the final concentrations of 0, 6.25, 12.5, 25, 50 and 100 μg·mL^−1^ just before the curdle of mediums, and then 5-mm discs of the growing fungus were inoculated at the center of 90 mm-diameter Petri plates, respectively. The plates were incubated at 25 °C and the radial growths were observed in 48 h intervals.

Osthol was added to Czapek liquor media (50 mL) in flasks to the final concentrations of 0, 6.25, 12.5, 25, 50 and 100 μg·mL^−1^, respectively. Each flask were inoculated with a 5-mm disc of *F. graminearum* and incubated at 25 °C in a rotary shaker of 140 rpm. Mycelium was harvested from each medium after 72 h, oven-dried, and the biomass was recorded.

At the same time, equal volumes of DMSO without osthol were also used as solvent controls in the same treatment. All experiments (including the following experiments) were repeated three times and each one experiment was done in triplicates.

### 3.3 Effect of osthol on hyphal morphology

The effects of osthol on the hyphal structure of *F. graminearum* were observed by light and transmission electron microscope (TEM). Osthol was added to incubation shaking flasks of *F. graminearum* to the final concentration of 100 μg·mL^−1^ three days after inoculation. Mycelium was harvested for morphology observation 24 hours after the treatment. For transmission electron microscopy (TEM), the samples were infiltrated, embedded with Epon-Araldite and polymerized at 60 °C for 24h. Ultrathin sections of the samples were cut with a diamond knife and collected on 200-mesh copper grids. After contrasting with uranyl acetate and lead citrate, the grids were examined with a HITACHI H-600 electron microscope at 75KV [18].

### 3.4 Preparation of hyphae

The pathogen *F. graminearum* was grown on potato dextrose agar (PDA) at 28 °C for 5 days and the 10 mm mycelial plates were inoculated into 500 mL flasks containing sterilized Czapek culture medium (250 mL). *F. graminearum* were cultured in flasks at 25 °C, 140 rpm for 72 h, and then were treated with osthol at a final concentration of 100 μg·mL^−1^. After 1, 3, 6, 12 and 24 h treatment, mycelial modality was observed separately. The harvested hyphal materials were stored in a refrigerator at −80 °C for the following trials.

### 3.5 Determining the mycelial of F. graminearum absorbed ^14^C-labelled glucose

Hyphae plates of *F. graminearum* grown on PDA medium were inoculated in Czapek culture medium in shake flasks and incubated at 25 °C, 140 rpm for 72 h and 15 μL 303.00 m Ci/mmol ^14^C-labelled glucose were added thereafter. Then the hyphae in the culture medium were treated with osthol at a final concentration of 100 μg·mL^−1^. After incubation for 1, 3, 6, 12 and 24 h, the hyphae were sampled and filtered through filter paper and stored at −20 °C. Hyphae from osthol-free mediums were used as control.

Scintillator solution: 5 g PPO (2,5-diphenyloxazole), 0.5 g POPOP (1,4-bis(5-phenyl-2-oxazolyl) benzene) were dissolved in toluene respectively mixed with 300 mL Triton X-100 and made up with toluene to a final volume of 1000 mL.

Accurately weighed 0.1 g freeze dried mycelial samples were homogenized by hand with Tris-HCl (0.05 M, pH=7.5, 1 mL), then the homogenate was centrifuged at 10000 rpm for 10 min at 4 °C. Supernatant (0.1 mL) was were mixed with scintillator solution (6 mL) and measured by scintillation counter. [19].

### 3.6 Determination of reducing sugar

For each treatment, mycelial material (0.4 g dry weight) was homogenized in extraction solution (0.05 M Tris-HCl, pH 7.5, 2 mL). The homogenate was centrifuged at 15000 rpm for 10 min at 4 °C, and the supernatant was used for the following trials. Supernatant (0.1 mL) was carefully transferred into an Eppendorf tube, and then DNS reagent (2.0 mL) was added to the ED tube. The tubes were heated at 100 °C in a water bath for 5 minutes, cooled down to room temperature and filled up to a predetermined volume of 25 mL. At the same time, distilled water (0.1 mL) plus DNS reagent (2.0 mL) were treated as described above and served as blank sample for zeroing of the spectrophotomater. Three repeats were carried out in each treatment and a glucose standard curve was prepared at a wavelength of 595 nm for calibration purposes [20].

### 3.7 Determination *of N-Acetyl-D-Glucosamine*

Supernatant liquid (0.2 mL) was treated with potassium borate solution (0.8 M, 0.1 mL) and the mixture was placed in a boiling water bath for 3 min. After cooling down, 1% DMAB (3 mL) was added and the samples were kept at 36 °C for 20 min. The absorbencies of the samples were detected at 544 nm using a spectrophotometer. Three repeats were carried out in each treatment and *N*-acetyl-D-glucosamine was used to make the standard curve [21].

### 3.8 Analysis of chitinase

Gelatiniform chitin (N-acetylation product of chitosan, 0.2 mL) was added to supernatant liquid (0.3 mL from 2.5) and the mixture were kept at 37 °C for 1 h and inactivated in boiling water for 5 min. After centrifugation at 5000 rpm for 10 min, supernatant liquid (0.2 mL) was kept in a boiling water bath for 3 min. After cooling down, potassium borate solution (0.8 M, 0.1 mL) and 1% DMAB (3 mL) were added. After incubation at 36 °C for 20 min, the values of absorbency for each treated sample were detected at 544 nm using a spectrophotometer. Three repeats were carried out in each treatment and *N*-acetyl-D-glucosamine was used to make the standard curve [22].

### 3.9 Analysis of β-1,6-glucanase activity

To supernatant liquid (0.3 mL from 2.5) 1% β-1,6-glucanase (0.4 mL) was added and the mixture was kept at 40 °C for 5 min, and then DNS (2 mL) was added. After incubation for 5 min in a boiling water bath and cooling down to the room temperature, the absorbance values for each treated sample were detected at 595 nm using a spectrophotometer. Three repeats were carried out in each treatment and glucose was used to make the standard curve [23].
